# Association of BMI, comorbidities and all-cause mortality by using a baseline mortality risk model

**DOI:** 10.1371/journal.pone.0253696

**Published:** 2021-07-09

**Authors:** Jia Li, Gyorgy Simon, M. Regina Castro, Vipin Kumar, Michael S. Steinbach, Pedro J. Caraballo

**Affiliations:** 1 Department of Computer Science and Engineering, University of Minnesota, Minneapolis, Minnesota, United States of America; 2 Department of Medicine, University of Minnesota, Minneapolis, Minnesota, United States of America; 3 Institute for Health Informatics, University of Minnesota, Minneapolis, Minnesota, United States of America; 4 Department of Medicine, Mayo Clinic, Rochester, Minnesota, United States of America; 5 Department of Health Science Research, Mayo Clinic, Rochester, Minnesota, United States of America; University of Zurich, SWITZERLAND

## Abstract

**Objective:**

The association of body mass index (BMI) and all-cause mortality is controversial, frequently referred to as a paradox. Whether the cause is metabolic factors or statistical biases is still controversial. We assessed the association of BMI and all-cause mortality considering a wide range of comorbidities and baseline mortality risk.

**Methods:**

Retrospective cohort study of Olmsted County residents with at least one BMI measurement between 2000–2005, clinical data in the electronic health record and minimum 8 year follow-up or death within this time. The cohort was categorized based on baseline mortality risk: Low, Medium, Medium-high, High and Very-high. All-cause mortality was assessed for BMI intervals of 5 and 0.5 Kg/m^2^.

**Results:**

Of 39,739 subjects (average age 52.6, range 18–89; 38.1% male) 11.86% died during 8-year follow-up. The 8-year all-cause mortality risk had a “U” shape with a flat nadir in all the risk groups. Extreme BMI showed higher risk (BMI <15 = 36.4%, 15 to <20 = 15.4% and ≥45 = 13.7%), while intermediate BMI categories showed a plateau between 10.6 and 12.5%. The increased risk attributed to baseline risk and comorbidities was more obvious than the risk based on BMI increase within the same risk groups.

**Conclusions:**

There is a complex association between BMI and all-cause mortality when evaluated including comorbidities and baseline mortality risk. In general, comorbidities are better predictors of mortality risk except at extreme BMIs. In patients with no or few comorbidities, BMI seems to better define mortality risk. Aggressive management of comorbidities may provide better survival outcome for patients with body mass between normal and moderate obesity.

## Introduction

The association between body weight distribution defined by body mass index (BMI = Kg/m^2^) and all-cause mortality is a controversial topic. Multiple studies, including several systematic reviews and meta-analyses, have attempted to explain this association and found different results [1–8]. The general association of BMI and all-cause mortality follows a U or J curve, with very high mortality among people with very low BMI (<18.5) and very high (BMI >40). However, the most common unexpected finding is that people defined as having a normal or ideal weight with BMI of 18.5 to 25 does not necessarily have the best survival. In many cases, overweight people (BMI 25 to 30), and those with mild to moderate obesity, (BMI of 30 to 35 and 35 to 40), show the best survival. This phenomenon has been described as the “obesity paradox” and it is the subject of intense review due to the potential and very significant impact on many aspects of routine clinical practice and the healthcare in general [9–24].

The obesity paradox has been described not only in the general population [1] but also in multiple cohorts of people with highly prevalent medical conditions including diabetes [22], heart disease [10, 25–27], kidney disease [28, 29], cancer [30, 31], stoke [32, 33], and rheumatoid arthritis [34], among others. The potential explanations are described sometimes in relation to the nature of the clinical condition, and sometimes attributed to methodological biases. For example, in patients with cancer and on chemotherapy, having more metabolic reserves may improve their chances of survival. However, in other conditions such as diabetes and congestive heart failure, obesity associated to better survival cannot be easily explained. Many possible methodological biases have been proposed, including reverse causation, confounding factors, and several biases such as selection, survival and treatment biases [22, 35]. However, these biases are not present consistently in all the studies.

Many discussions also center on the definition of normal weight and obesity by using BMI [36, 37]. Experts agree that BMI is an imperfect measure of body fat and may be influenced by many factors, including body composition of muscle mass, fat distribution, visceral vs. subcutaneous fat, and ectopic fat. Even more important, physical fitness and nutritional status may play a more important role than BMI in predicting overall health and risk of mortality [38]. However, one concept seems to be well accepted, obesity is a well-established and important risk factor for many metabolic and cardiorespiratory conditions, and these conditions have been associated with reduced survival [39]. Now, the question raised by this paradox is: In the presence of one or more clinical conditions, and once modern therapeutic interventions have been taken to manage these underlying metabolic consequences of obesity, is BMI still a main determinant of survival? Are there other factors in the complex relation between diseases, therapy and follow-up that become more important than the BMI?

Based on these observations, the aim of this study was to use a new approach to assess the relationship between BMI and all-cause mortality by using a risk model based on multiple clinical characteristics, including comorbidities, to define baseline mortality risk and analyze the association between this mortality risk and BMI using the BMI in categories and as a continuous variable.

## Methodology

### Study setting and participants

Retrospective cohort study conducted using clinical data from the Mayo Clinic electronic medical records and related databases including the Rochester Epidemiology Project (REP). Mayo Clinic is a large integrated Medical Center located in Rochester, Minnesota, that provides care to national and international patients but also to the Rochester community. It has an integrated, inpatient and outpatient, electronic medical record (Epic, Madison, WI) and comprehensive electronic clinical data dating since the 1990’s The REP is a research data repository approved for medical research. It links together almost all the medical records of the residents of Olmsted County, MN, over several decades [40, 41]. From this repository, electronic searches identified a cohort of 52,148 Olmsted county residents seen at Mayo Clinic in Rochester, Minnesota, between January 1, 2000 and January 1, 2005. This cohort included adults aged 18 or older, alive on 2005-01-01, and with at least one BMI measurement between 2000 and 2005. Median follow-up was 9.2 years. Subjects that dropped out without an event within 8 years and those for whom 8-year mortality could not be calculated were excluded leaving 39,739 subjects in the study cohort. According to Minnesota state law (Minnesota state privacy law, Statute 144.335), subjects without research authorization were excluded. The study followed best practice to protect the confidentiality of the medical data, including those related to the Health Insurance Portability and Accountability Act (HIPAA). This study was reviewed and approved by Olmsted Medical Center and Mayo Clinic Institutional Review Boards.

### Data collection

Data were collected using electronic search of the REP database and the Mayo Clinic electronic health record including inpatient and outpatient visits. The baseline data was collected between January 1, 2000 and January 1, 2005 and included demographic data, vital signs, laboratory results, medications and diagnoses. We collected height, weight and calculated BMI that were measured during clinic visits and documented in the electronic medical records. Multiple BMI observations were aggregated by computing their average. Prescriptions between 2003–2005 were collected and aggregated using NDF- RT therapeutic classes (antidiabetic, antihypertensive and antihyperlipidemic drugs). We used the Elixhauser Comorbidity list to categorize the comorbidities of the patients using the International Classification of Diseases (ICD) diagnosis codes found in the EHR. Each comorbidity category was considered either present or not present (dichotomous) [42]. The primary outcome was all-cause mortality. Mortality-status was collected using the REP database during the entire follow-up period and allowed to calculate 8-year mortality rates [41].

### Mortality risk model and risk groups

We constructed a risk model to assess the individual 8-year all-cause mortality risk at baseline, excluding the direct effect of obesity for all the subjects in the cohort. A logistic regression model was used with independent variables including age, sex, Exlihauser Comorbidities except obesity, and additional variables including hyperlipidemia, coronary artery disease, chronic kidney disease, and stroke. There were no missing values and backwards elimination was used for feature selection. Only hyperlipidemia showed opposite effect due to collinearity with coronary artery disease. The risk model achieved an area under the receiver operating characteristic curve (AUC) of 0.91.

The cohort was categorized in five risk groups using percentiles of the patient’s baseline mortality risks derived from the model:

1. Low: patients in the lower half of the risk scores.
2. Medium: patients between 50th and 75th percentile of the risk scores.
3. Medium-high: patients between 75th and 90th percentile.
4. High: patients between the 90th and 95th percentile.
5. Very-high: patients above the 95% percentile.

Higher risk groups had much more comorbidities than lower risk groups. The low risk group had on average 0.8 comorbid conditions, the medium risk group had 2, the medium-high had 3.6, the high risk group had 5.1 and the very-high risk group had 7.8 and an approximately 80% 8-year mortality rate.

### Body mass index

The average BMI for each individual at baseline was used in the study. BMI categories were defined every 5 points starting at <15 and ending at ≥45. These categories differ from those defined by the National Institutes of Health and the World Health Organization in the categories with cutoff of 18.5, underweight <18.5 and normal 18.5 to <25. These categories allowed the analysis to avoid preconceptions related to what should be normal or abnormal (underweight, normal and obesity) [36, 43]. The BMI was also used as a continuous variable with intervals of 0.5 Kg/m^2^.

### Statistical analysis

Unadjusted mortality rate was calculated as the fraction of patients who died within 8 years among patients in the given risk group and BMI category. To better understand the effect of BMI on mortality, BMI was also used as a continuous variable at 0.5 kg/m^2^ resolution. For each BMI level, between 15 and 55, we computed the unadjusted mortality rate using kernel estimation with a Gaussian kernel. The empirical 95% confidence interval was obtained through 200 iterations of bootstrap resampling.

To adjust for baseline risk and to assess whether some of the excess mortality risk could be attributed to BMI, we considered residuals. For a patient, the residual was the difference between the patient’s predicted risk and the actual outcome. It could be interpreted as the patient’s excess risk of mortality not explained by the baseline comorbidities. As before, for each BMI level between 15 and 55 we computed the average residual (excess risk of mortality) using kernel estimation with a Gaussian kernel and bandwidth of 0.5 kg/m^2^. The empirical 95% confidence interval was obtained through 200 iterations of bootstrap resampling.

## Results

### Baseline characteristics and risk groups

The cohort of 39,785 adult subjects (average age 52.6, range 18–89; 38.1% male) had an overall all-cause mortality of 11.9% (4,713 deaths) in 8-year follow-up.

The mortality risk model was used to define five risk groups (Low, Medium, Medium-high, High and Very-high) as defined in the methodology. Table 1 shows the baseline characteristics of the entire cohort and the risk groups. The mean age and the prevalence of the comorbidities increased with the increase in the mortality risk in all the risk groups.

**Table 1:** Baseline comorbidities and mortality risk model.

Comorbidities	Prevalence of Comorbidities in the Risk Groups (%)						Mortality Risk Model	
	Low	Medium	Medium High	High	Very High	Entire Cohort	Coefficient	p-value
N	19865	9939	5961	1987	1987	39739	-	-
(Intercept)	-	-	-	-	-	-	-8.112	<0.001
Age (years, mean)	38.1	58.1	72.0	80.9	83.0	52.6	0.082	<0.001
Male (%)	31.8	43.0	45.9	43.3	47.1	38.1	0.231	<0.001
Hyperlipidemia	24.8	49.3	62.2	57.9	53.5	39.6	-0.584	<0.001
Hypertension	13.6	44.3	70.6	82.4	89.3	37.1	0.132	0.007
Chronic lung disease	13.7	20.4	26.2	33.9	46.5	19.9	0.452	<0.001
Deficiency anemia	6.1	11.2	23.1	36.5	61.4	14.2	0.232	<0.001
Psychoses	9.2	11.9	9.9	13.7	27.1	11.1	0.372	<0.001
Fluid and electrolytes disorder	3.0	6.2	16.2	33.7	65.4	10.5	0.555	<0.001
Coronary artery disease	1.4	11.6	31.5	46.3	62.0	13.7	0.342	<0.001
Valvular disease	2.7	7.2	17.7	29.1	46.0	9.5	0.196	0
Other neurological disorders	2.2	6.1	11.9	21.9	42.0	7.6	0.754	<0.001
Diabetes mellitus	2.7	11.7	22.0	26.1	33.8	10.5	0.442	<0.001
Peripheral vascular disorder	0.7	3.8	13.7	24.0	37.3	6.4	0.410	<0.001
Solid tumor without metastasis	1.4	6.3	16.1	23.0	30.0	7.4	0.400	<0.001
Weight loss	1.4	2.3	4.7	9.6	21.5	3.6	0.562	<0.001
Congestive heart disease	0.1	0.7	5.2	19.3	52.6	4.6	0.860	<0.001
Alcohol abuse	2.2	4.3	4.9	4.1	5.6	3.4	0.622	<0.001
Chronic kidney disease	0.2	0.8	3.2	7.4	21.9	2.2	0.612	<0.001
Pulmonary circulation disorder	0.4	1.0	4.1	8.5	18.8	2.4	0.291	0.002
Coagulopathy	0.8	1.6	3.9	6.5	14.9	2.4	0.485	<0.001
Liver disease	1.0	2.6	3.7	3.4	4.8	2.1	0.507	<0.001
Stroke	0.1	0.7	3.2	7.1	15.7	1.8	0.251	0.019
Drug abuse	1.6	2.2	2.0	2.1	2.1	1.8	0.755	<0.001
Metastatic cancer	0.0	0.9	3.4	7.0	12.4	1.7	1.212	<0.001
Paralysis	0.3	1.0	2.3	4.0	11.1	1.5	0.744	<0.001
Lymphoma	0.2	0.6	1.9	2.4	3.9	0.8	0.588	0
HIV/AIDS	0.0	0.0	0.2	0.2	0.1	0.0	1.810	0.003

Comorbidities with prevalence less than 1% were omitted in this table.

**Fig 1:**
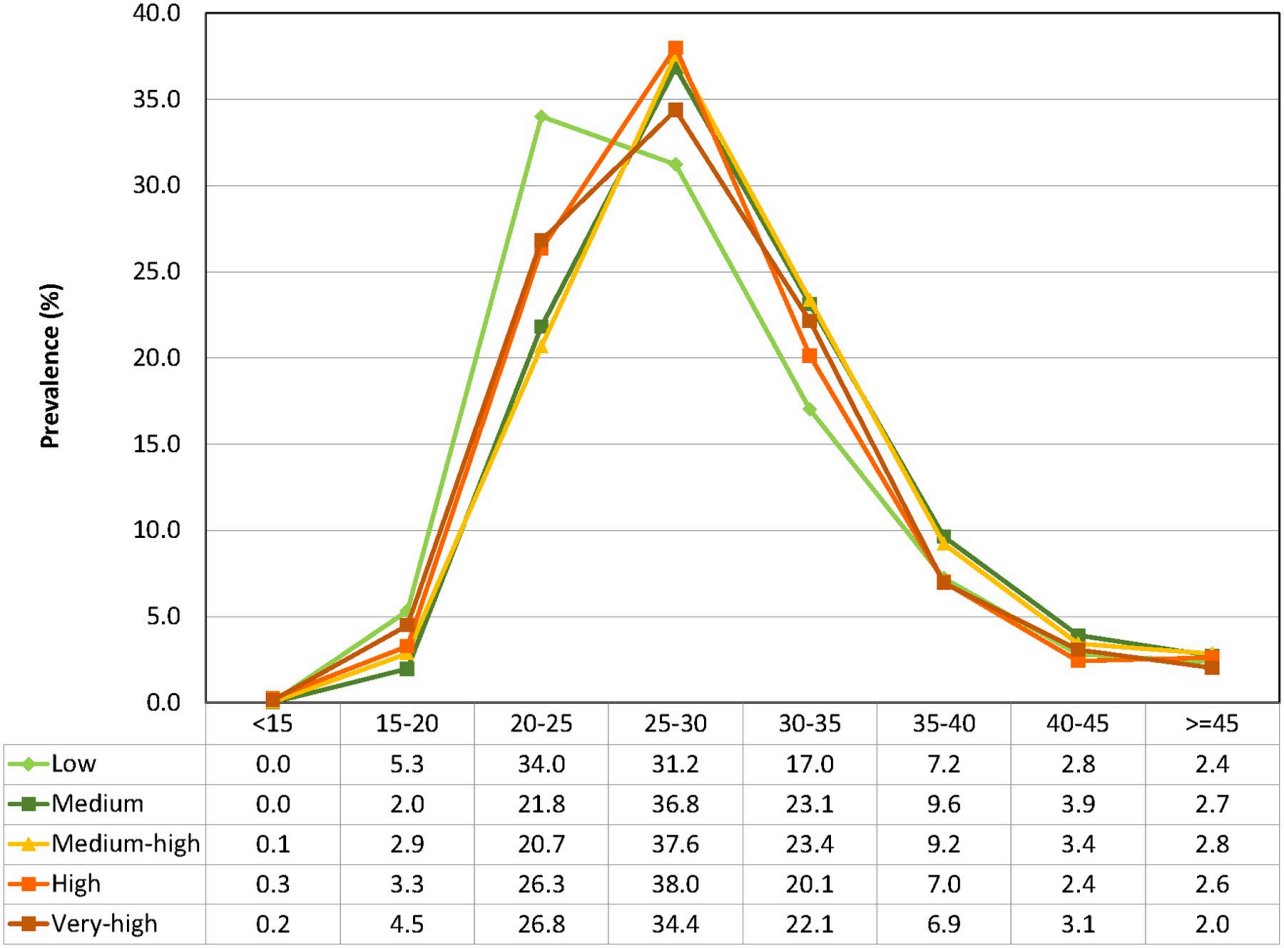
Distribution of the prevalence (%) of the eight BMI categories among the five mortality risk groups: Low, medium, medium-high, high and very-high risk.

The distribution of BMI within each risk group was very similar except for the low risk group that had slightly more subjects with BMI 20 to <25 and slightly less with BMI 25 to <35 (Fig 1). The category with the most subjects was BMI between 20 and 35 with 13,543 subjects. The number of subjects with BMI <15 was small (n = 22), followed by BMI ≥45 (n = 1000). The rest of the BMI categories had between 1,259 and 13,543 subjects (Table 2).

**Table 2:** Raw 8-year mortality rates in the entire cohort and the risk groups by BMI categories.

BMI	Entire Cohort			Mortality Risk Groups				
	N = 39739	Mortality N = 4713	Mortality Rate (0.1186)	Low	Medium	Medium High	High	Very High
<15	22	8	0.364	0.000	0.000	0.333	0.800	1.000
15–<20	1576	243	0.154	0.009	0.108	0.453	0.785	0.944
20–<25	11212	1193	0.106	0.006	0.040	0.278	0.581	0.784
25–<30	13543	1652	0.122	0.012	0.043	0.222	0.514	0.782
30–<35	7909	989	0.125	0.011	0.044	0.226	0.513	0.752
35–<40	3218	349	0.108	0.006	0.039	0.239	0.496	0.746
40–<45	1259	142	0.113	0.014	0.057	0.225	0.521	0.672
>=45	1000	137	0.137	0.021	0.074	0.266	0.577	0.800

BMI = body mass index, Kg/m^2^.

### Mortality risk among the risk groups based on BMI

Table 2 shows the 8-year mortality rates in each BMI category for the entire cohort and each risk group. The categories with very low BMI and very high BMI showed the highest mortality rates for all the risk groups. The intermediate categories showed lower risk, picturing the traditional “U” distribution for all the risk groups but with a wide and plateau nadir.

The changes in mortality rates between risk groups were more significant compared to changes between BMI categories within the same risk group. For example, for BMI 25 to <30 the mortality rate for the Low, Medium, Medium-high and High risk groups is 0.012, 0.043, 0.222 and 0.514 respectively. However, in the Medium-high risk group the mortality rate for the BMI of 25 to <30, 30 to <35 and 35 to <40 was 0.222, 0.226 and 0.239 respectively.

**Fig 2:**
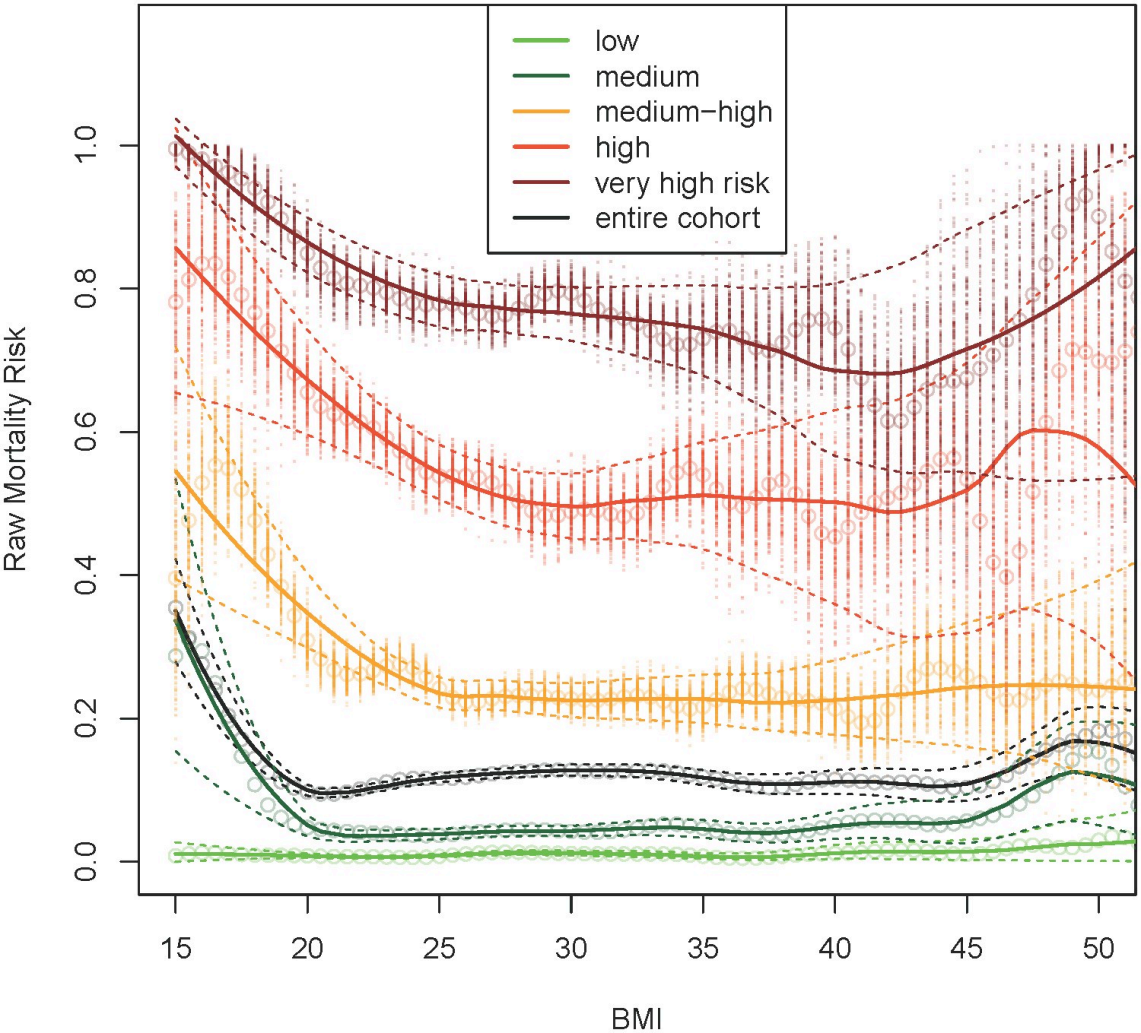
Raw 8-year mortality rates at each 0.5 Kg/m^2^ BMI intervals between 15 and 55 in the entire population (black line) and the five mortality risk groups. Dots represent the point estimates of the mortality rate in the 200 bootstrap iterations, circles are their mean, the solid line is a smooth curve representing the averages and the dashed lines are the 95% empirical confidence interval.

These findings are better appreciated in Fig 2 where each risk group and the entire cohort are represented as their unadjusted mortality risk and 95% confidence interval, using BMI as a continuous variable. The mortality risk increases significantly between the risk groups, with relatively small changes attributable to changes in BMI, except for very low and very high BMI. In these extreme BMI categories, the mortality risk seems to have a noticeable increase but with wide confidence intervals. The nadir for each group is a plateau, except for the high and very high risk groups that have fluctuations in mortality risk in the BMI over 35 and 40. Mortality seems to be low in the Very-high risk group with BMI close to 40. These values are difficult to interpret and likely are a statistical anomaly evident by the wide confidence intervals that denote the small and likely heterogeneous group of subjects.

### Effect of body mass index on mortality adjusted by comorbidities

**Fig 3:**
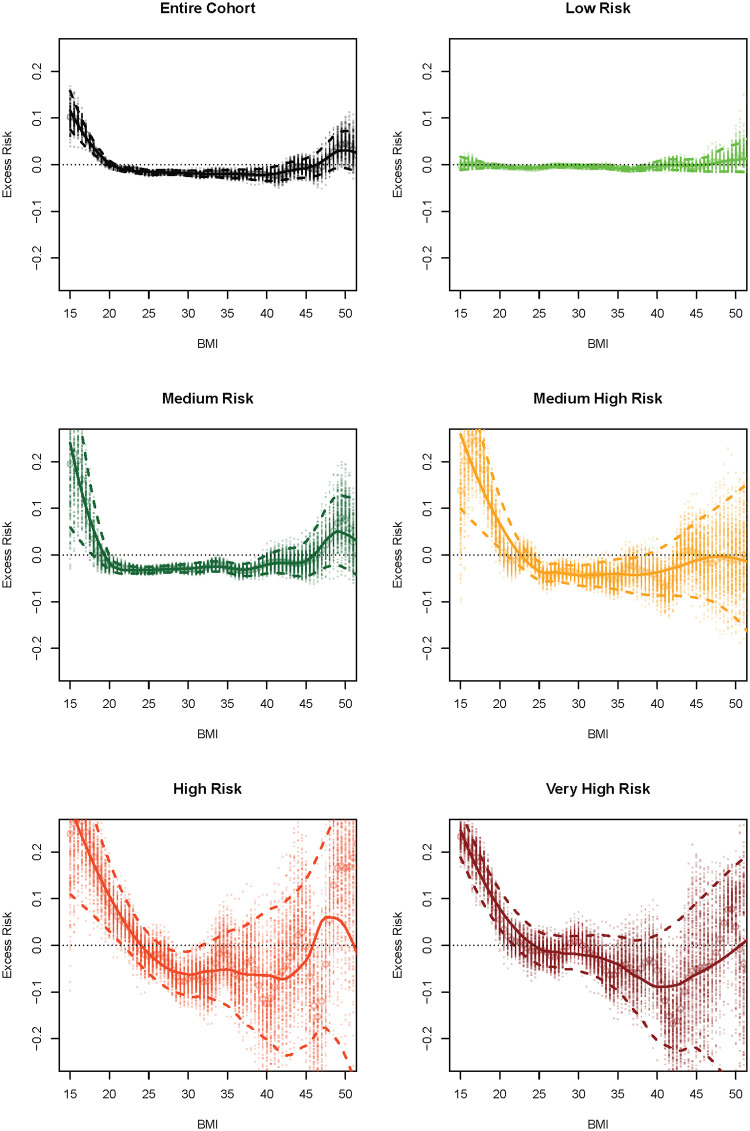
Excess risk of all-cause mortality attributed to the BMI not explained by the baseline comorbidities (residual risk) after adjusting for baseline mortality risk. The residual risk was estimated for each 0.5 Kg/m^2^ BMI interval between 15 and 55. The dots represent the point estimates of the mortality rate in the 200 bootstrap iterations, circles are the mean, the solid line is a smooth curve representing the averages and the dashed lines are the 95% empirical confidence interval. Values above zero represent increase risk and values below zero represent decreased risk compared to the group average.

Fig 3 shows the excess risk of mortality as a function of BMI as a continuous variable (0.5 kg/m^2^ between 15 and 55) for the entire cohort and each risk group. The residual mortality risk that is not explained by the comorbidities but attributed to BMI has again a “U” distribution with a plateau nadir for the entire cohort and all the risk groups. For all the groups, low BMI and very high BMI were associated with mortality higher than the average of the risk group (residuals are consistently above the zero line). Conversely, intermediate BMIs were associated with a lower mortality than the average of the risk group (residuals were consistently below the zero line). This distribution was easy to appreciate in the entire cohort and the Low, Medium and Medium-high groups. In the High and Very-high groups this trend is less clear, in part due to the low number of subjects and the consequent wide confidence intervals. Also, the low risk does not seem to correspond to the traditional definitions of normal, overweigh and obesity. For example, for the entire cohort, BMI approximately between 20 and 40 showed similar residual mortality risk when adjusted by comorbidities.

## Discussion

Our results show that the main driver of all-cause mortality risk is comorbidities, assessed in our study by a risk model mainly based on baseline comorbidities and the categorization of the risk in five mortality risk groups. While moving to a higher comorbidity-based risk group (by developing new comorbidities) increases the mortality risk by approximately 20–30% (except between low and medium risk groups), the interquartile variation within the risk groups is much smaller when we exclude extreme low and extreme high BMI.

In general, within each risk group, the distribution of the BMI was close to a normal distribution and very similar among all the groups. The association between BMI and all-cause mortality displayed “U” distribution regardless of the baseline mortality risk. Our data did not show a nadir in a specific BMI category; instead the nadir was in general a plateau including what is defined as normal, overweight and obesity with some variations across the different risk groups. These findings suggest that the mortality risk is essentially flat across these BMI categories. Patients with extreme BMI (underweight and extreme obesity) show increased risk of mortality, independent of the baseline comorbidity risk, but the (interquartile) variation within a risk group is much smaller than the risk difference between groups.

Subjects in the low and medium risk groups, which comprise 75% of the population, have very few or no comorbidities. In the absence of serious comorbidities in these patients, mortality depends primarily on BMI. In particular, BMI in excess of 38 kg/m^2^ is associated with increased mortality. In low risk patients, the increase in mortality between 25 and 40 kg/m^2^ is a relative 65% (from 0.8% to 1.10%). Similarly, in medium risk subjects, the increase in mortality risk between 25 and 40 kg/m^2^ is 68% (from 3.6% to 5.3%). In the absence of significant comorbidities, BMI is an important driver of mortality; however, the variation of mortality risk based on BMI changes within each group is much smaller than the risk difference between the groups.

We also encountered some paradoxical findings, but only in the top 5% of the population with the most baseline comorbidities. These patients have 78% mortality in 8 years on average. In these subjects, and only in these subjects, obesity with BMI <42 may be protective, but BMI in excess of 42 kg/m^2^ is still associated with increased mortality. However, these data should be interpreted cautiously, given the small number of subjects and wide heterogeneity represented by the wide confidence intervals.

The stark contrast between the lack of correlation between all-cause mortality and BMI categories defined as normal, overweight and obesity, and the strong association between the baseline comorbidities and all-cause mortality has significant clinical relevance. Obesity is an important risk factor for many chronic and common clinical conditions; however, once the conditions are overt, the mortality risk seems to be more closely correlated with the prevalence and management of these conditions than with the body weight except for extremes values. Obesity, measured by BMI, is a readily available clinical indicator used across the health care system triggering additional clinical interventions. However, our data showed that the BMI distribution is very similar among all the risk groups, which should reinforce the premise to screen and manage comorbidities regardless of the BMI.

The obesity paradox has been observed and reported in several common conditions including diabetes, coronary artery disease, hypertension, etc., for which BMI is an important known risk factor [15, 22, 44]. The cause of these observations requires additional evaluation that needs to be specific to each condition, considering not only additional comorbidities, but also temporal metabolic and therapeutic changes that could potentially lend a survival advantage, as long as the BMI is not extreme. The purpose of this work is neither to disprove the existence of such an effect nor to dismiss BMI as a clinical risk factor, but to convey the need for aggressive management of comorbidities regardless of BMI. Patients with low BMI may look healthier, which in turn may lead to being less aggressive in screening for or treating comorbidities. For example, high BMI has been identified as an independent predictor of recognition and management of prediabetes while low BMI predicted lack of recognition [45]. The development of new comorbidities imparts a far greater impact on mortality risk than the variation of BMI; thus, prevention, recognition, and management of comorbidities should be of high priority, regardless of BMI.

Our findings summarize a complex relationship between BMI and all-cause mortality and may not solve current controversy. However, our findings are in keeping with results of many other studies that describe this complex relationship that cannot be attributed to any single statistical bias or limitation of the BMI as a definition of obesity. We must become critical of the current definitions of ideal body mass and what is or is not abnormal or related to health risks. Only reevaluating our current premises, we will be able to find evidence (no expert opinions) that can be implemented in daily clinical practice.

All the primary limitations of an observational retrospective cohort study should be considered in our study. The data collection was done using electronic search which allowed the use of a large data set but inevitably decreases the accuracy and details of individual variables. One relevant variable that was a casualty of our electronic searches was smoking status. Smoking is associated to lower BMI and higher mortality and it is well known as a risk factor for several comorbidities including cancer, cardiovascular and lung diseases, which were included in our prediction model. Unfortunately, smoking data are usually recorded as text in the clinical notes but lack of standardization and reliability. The limited structured data available in the medical record were contradictory and found to be too inaccurate to be used in the model. However, for other relevant variables, we used data collected during routine clinical care and stored in the EHR and other data repositories intended for research. Still, our study uses data from a defined cohort seeking care in one specific medical center which may limit generalizability and our findings should be subjected to validation. This is not a population-based study and likely healthy individuals are underrepresented. Our results should be used in the context of clinical care, especially in subjects with comorbidities.

In conclusion, our study shows that baseline comorbidities, through the risk they confer on patients, are the primary driver of all-cause mortality, while BMI only plays a secondary role. In general, across all baseline comorbidity-based risk groups, the association between BMI and all-cause mortality displayed U-shaped distribution. The nadir was a plateau including what is defined as normal, overweight and obesity with variations across the different risk groups. Patients with extreme BMIs within each risk group showed increased risk of mortality, but the variation of mortality risk within each group was smaller than between groups. This suggests that patients should be treated aggressively to prevent or manage comorbidities, regardless of BMI.
